# Detection of Infectious Agents Causing Neonatal Calf Diarrhea on Two Large Dairy Farms in Yangxin County, Shandong Province, China

**DOI:** 10.3389/fvets.2020.589126

**Published:** 2021-02-05

**Authors:** Xiaojuan Wei, Weiwei Wang, Zhen Dong, Fusheng Cheng, Xuzheng Zhou, Bing Li, Jiyu Zhang

**Affiliations:** ^1^Lanzhou Institute of Husbandry and Pharmaceutical Sciences of Chinese Academy of Agricultural Sciences (CAAS), Lanzhou, China; ^2^Key Laboratory of New Animal Drug Project of Gansu Province, Lanzhou, China; ^3^Key Laboratory of Veterinary Pharmaceutical Development, Ministry of Agriculture, Lanzhou, China

**Keywords:** neonatal calf diarrhea, etiological agents, China, cattle, Yangxin County

## Abstract

Neonatal calf diarrhea (NCD) is one of the most serious health challenges facing the livestock industry and has caused substantial economic losses due to increased morbidity and mortality rates. The present study investigated the main infectious pathogens causing NCD among cattle in Yangxin County, China. Sixty-nine fecal samples were collected from diarrheic newborn cattle and tested for infectious agents, including bovine rotavirus, bovine coronavirus, *Escherichia coli* K99, *Cryptosporidium parvum*, and *Giardia lamblia*, that cause NCD, as determined by rapid kit analysis and polymerase chain reaction (PCR) amplification. The PCR results showed that the percentages of samples that were positive for *C. parvum*, bovine rotavirus A, bovine coronavirus, and *G. lamblia* were 44.93, 36.23, 17.39, and 13.04%, respectively. The rapid kit analysis results showed that the prevalence of *C. parvum*, rotavirus, coronavirus, and *G. lamblia* was 52.17, 31.88, 28.98, and 18.84%, respectively. No *E. coli* K99 was detected by either method. The total positivity of the samples, as determined by PCR and rapid kit analysis, was 80.00 and 81.16%, respectively. No significant difference between the two methods was observed. The results of this study may help to establish a foundation for future research investigating the epidemiology of NCD in cattle and may facilitate the implementation of measures to control NCD transmission to cattle in Yangxin County, Shandong Province, China.

## Introduction

Neonatal calf diarrhea (NCD) is one of the most serious diseases worldwide among newborn calves (<1 month old). NCD causes notable levels of morbidity and mortality through several complications, such as dehydration, acidosis, and solution depletion (1–3). At present, NCD continues to be a major cause of fruitfulness reduction and economic loss in *Bos taurus* herds around the world (4), despite advances in the cattle industry regarding herd management, animal facilities and care, feeding and nutrition, and timely use of biopharmaceuticals. This disease may be triggered by both infectious and non-infectious factors (the intrinsic characteristics of the calf, its organic processes, veterinary treatment, management of the herd, and environmental factors) (5). Among these factors, infectious agents are the leading cause of death. Among the numerous infectious agents causing NCD, rotavirus, coronavirus, *Escherichia coli* enterotoxin K99/F5, and *Cryptosporidium parvum* spp. are recognized as the four most essential pathogens (6). *C. parvum* and rotavirus are often detected in fecal samples (2). In past research, most analyses centered on individual pathogens, but recent studies have urged that concurrent infection of a number of pathogens may be necessary to model the pathophysiology of gastrointestinal diseases (7). A study reported that the rate of coinfection was 55% in fecal samples obtained from diarrheic calves. Notably, the rate of coinfection in healthy calves was only 3% (8). Cryptosporidiosis is a worldwide zoonotic disease, and cattle are recognized as a major parasite reservoir and contributor to zoonotic infection (9). Therefore, research into the pathogens that infect cattle and cause NCD is vital for farmers and staff members in regions with intensive cattle farming. On the other hand, antibiotics are currently measured through empirical observation based on when the symptom of loose stools occurs, though a proper etiological diagnosis is rarely made, and it may not be clear what the cause is. This approach leads to the unnecessary and excessive use of antibiotics in food animal species and the potential development of antibiotic-resistant bacteria. This phenomenon has prompted a ban on the subtherapeutic usage of antibiotics in several countries (10).

The aim of this study was to detect the types of pathogens causing NCD and to determine the prevalence of this disease in two intensive cattle herds in Yangxin County, Shandong Province, China, through diagnosis in order to design rational and efficacious protocols for the prevention and treatment of diarrhea.

## Materials and Methods

### Sampling

**Figure 1:**
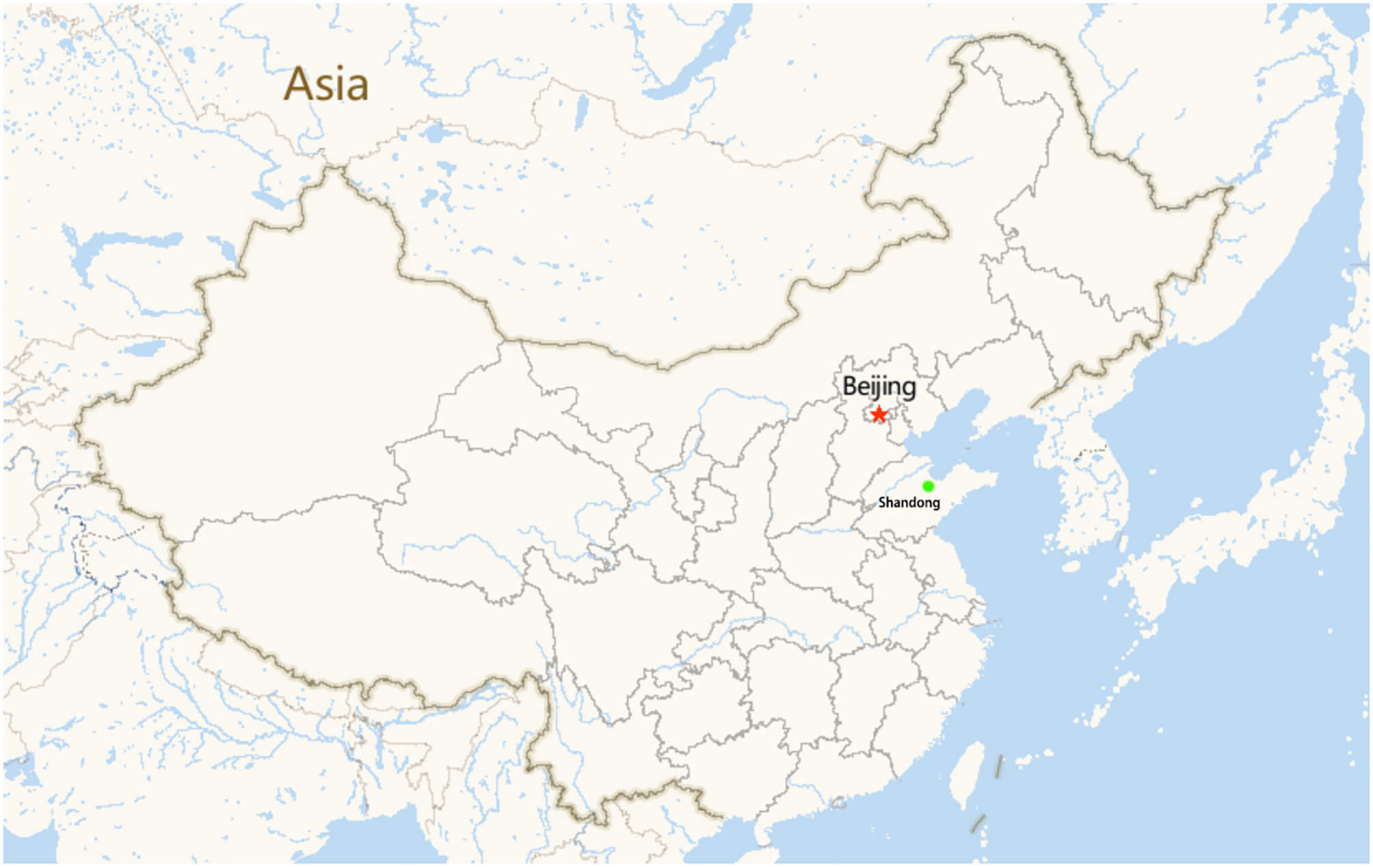
Small geographical map of the farms (the green dots represent the area where the cattle farms are located, the red star represent the capital of China, Beijing).

The study area was conducted in Yangxin County, Shandong Province, eastern China. This area is located between north latitudes 37°58′ and 37°66′ and east longitudes 117°47′ and 117°77′. A small geographical map of the farm is shown in Figure 1. This area is one of the most important cattle and dairy farming areas in the country. A total of 69 fresh stool samples were collected on October 8–10, 2019. At the beginning of October, during the NCD outbreak on the farm, the clinical signs were depression, anorexia, dehydration, and watery diarrhea. When the calves were administered broad-spectrum antibiotics (such as florfenicol), the animals were unresponsive to these medicines. Death occurred in four calves 3–5 days after the onset of diarrhea. To confirm the causes of the infectious agents, fresh stool specimens were obtained from calves for <1 month. Fifteen calves with no abnormal clinical signs were employed as controls, and 54 diarrheic calves were employed as the experimental group.

The female cows are transferred to the maternity barn after delivery, and the newborn calves are transferred to the calving barn in time. The colostrum is milked within 6 h after delivery, checked for quality, pasteurized, and then frozen. All calves were fed 4 L of colostrum within 12 h after birth. Calves were housed centrally in housing with ventilation, natural temperature, and unheated conditions. The study was conducted at two dairy cow farms (the average number of cows and breeding heifers is 800, with an average milk yield of 25 kg per cow per day). Heifers were not vaccinated against coronavirus and rotavirus. The cows were housed in a cattle-dense feedlot with all feed provided in a feed bunk and in individual pens. The amount of pasteurized whole milk offered to calves per day was about 6 L. Fecal samples were collected using disposable sterile plastic straw trimmed into the shape of a shovel and temporarily stored thereafter in an aseptic plastic sampling tube. To ensure that samples were not disturbed by microbes in the environment, swabs were utilized to take parts of the fecal center when sampling. All samples were stored in liquid nitrogen and transported to the laboratory for research. The numbers and ages of the animals were noted on each sampling tube.

The study obtained consent from the dairy farmers when collecting samples.

### Detection of Infectious Agents by Anigen Test Kit

Stool samples were tested using the Anigen Rapid BoviD-5 Ag Test Kit (BioNote, Korea) within a short period after collection on the farm. The Anigen Rapid BoviD-5 Ag Test Kit employs a solid-phase immunochromatographic assay for the rapid and qualitative detection of *C. parvum* antigen, bovine rotavirus antigen, bovine coronavirus antigen, *E. coli* K99 antigen, and *Giardia lamblia* antigen in feces. The testing process was performed in accordance with the manufacturer's instructions.

### DNA Extraction

The stool genomic DNA of feces was extracted using the E.Z.N.A. Stool DNA Kit (Omega, Georgia, USA). The genomic DNA was subsequently utilized to detect *G. lamblia, C. parvum*, and *E. coli* K99 by polymerase chain reaction (PCR) amplification.

### RNA Extraction

The clinical fecal samples were diluted with phosphate-buffered saline (PBS), vortexed, and centrifuged at 13.8 *g* at 4°C for 5 min. The supernatant was collected and used to extract viral RNA. Total RNA from feces was extracted using a Viral RNA Kit (OMEGA, Georgia, USA) according to the instruction of the manufacturer. The viral RNA was subsequently used as a template for reverse transcription (RT)-PCR.

### Confirmation of Infectious Agents by PCR Sequencing and Analysis

Primers for *C. parvum, G. lamblia, E. coli*, bovine rotavirus, and bovine coronavirus were used to detect infectious agents, as shown in Table 1.

**Table 1:** Primers used for PCR.

**Pathogen species**	**Primer**	**Sequence (5′-3′)**	**Product size (bp)**	**References**
*C. parvum*	SSU-F1 SSU-R1	TTCTAGAGCTAATACATGCG′ CCCTAACCTTCGAAACAGGA	587	(11)
	SSU-F2 SSU-R2	GGAAGGGTTGTATTTATTAGATAAAG CTCATAAGGTGCTGAAGGAGTA		
*G. lamblia*	Gia2029 Gia2150	AAGTGTGGTGCAGACGGACTC CTGCTGCCGTCCTTGGATGT	497 292	(12, 13)
	RH11 RH4	CATCCGGTCGATCCTGCC AGTCGAACCCTGATTCTCCGCCAGG		
	G7-F 759-R G376-F	AAGCCCGACGACCTCACCCGCAGTGC AGGCCGCCCTGGATCTTCGAGACGAC ATAACGACGCCATCGCGGCTCTCAGGAA	753 384	(14, 15)
	GDHeF GDHiRm GDHiF	TCAACGTYAAYCGYGGYT TCCGT GTTRTCCTTGCACATCTC CAGTACAACTCYGCTCTCGG	432	(16)
	GGL-F GGL-R	AAG TGC GTC AAC GAG CAG CT TTA GTG CTT TGT GAC CAT CGA	171	(17)
*E. coli* K99	ompA-F ompA-R	AGCTATCGCGATTGCAGTG GGTGTTGCCAGTAACCGG	919	(18)
Bovine rotavirus	VP7-F VP7-R	GGCTTTAAAAGCGAGAATTTCC GGTCACATCATACAACTCTAAT	1,062	(19)
Bovine coronavirus	BCV-P1 BCV-P2	GAGCGTCCTTTGGAAATCGT GCTTAGTTACTTGCTG TGGC	730	(20)

Nested PCR was performed to detect *C. parvum* and *Giardia* using 2× Taq Master Mix (Vazyme, Nanjing, China). PCR amplification of *E. coli* was carried out using 2× ExTaq DNA polymerase (Vazyme, Nanjing, China).

Fragments of the 16S rRNA gene were amplified. A nested PCR protocol was used with the first-round primers Gia2029 and Gia2150 and the secondary primers RH11 and RH4; the β*-giardin* gene was amplified by nested PCR using G7/G759/G376 primers and PCR by GGL/GGR primers; the *gdh* gene was amplified by nested PCR using GDHeF/GDHiRm/GDHiF primers.

All PCR products were visualized on a 1.5% agarose gel. All positive samples were purified and submitted for Sanger sequencing (Tianqigene Bio Com, Lanzhou, China). The sequence results were aligned in GenBank for each possible *C. parvum* species, *G. lamblia*, and *E. coli*.

One-step RT-PCR was performed to confirm bovine rotavirus and bovine coronavirus using the PrimeScript One Step RT-PCR Kit, Ver. 2 (TaKaRa, Dalian, China). The reaction was performed with an initial reverse transcription step at 50°C for 30 min, followed by PCR amplification at 94°C for 2 min and 30 cycles (98°C for 10 s and 68°C for 1 min). PCR products were visualized on 1.0% agarose gels, and positive samples were subsequently selected for sequencing. The sequence results were compared using BLAST in GenBank to confirm the virus species.

### Statistical Analysis

Statistical analysis was performed with R software, version 3.5.3. Chi-square tests were performed at a 5% level of significance.

## Results

### Detection of Infectious Agents by Rapid Kit Analysis

In this study, four etiological agents were tested, namely, *C. parvum*, rotavirus, coronavirus, and *G. lamblia*. From 69 fecal samples, 56 samples (81.16%) were positive for infectious agents. The results of the experiment are shown in Tables 2, 3 and Supplementary Figures 1, 2. The incidences of a single etiological agent with positive samples were as follows: *C. parvum* in 30.43% and *G. lamblia* in 8.70%. Negative samples were 20.29% (14/69). The rest of the samples exhibited coinfection.

**Table 2:** Number of infectious agents detected by two different analysis methods.

**Agent**	**Positive number and percent (*****n*** **= 69)**		**P (***χ***^2^)**
	**Rapid kit analysis**	**PCR**	
*C. parvum*	36 (52.17%)	31 (44.93%)	0.41 (0.67)
Bovine rotavirus	22 (31.88%)	25 (36.23%)	0.48 (0.50)
Bovine coronavirus	20 (28.98%)	12 (17.39%)	0.16 (1.99)
*G. lamblia*	13 (18.84%)	9 (13.04%)	0.26 (1.28)
Total positive	56 (81.16%)	60 (80.00%)	0.84 (0.64)

**Table 3:** Detection results of infectious agents by rapid kit analysis.

**Agent**	**Number**	**Percent**
None	14	20.29%
*C. parvum*	21	30.43%
*C. parvum* + rotavirus	8	11.59%
*C. parvum* + coronavirus	2	2.90%
*C. parvum* + rotavirus + coronavirus	4	5.80%
*C. parvum* + rotavirus + coronavirus + *G. lamblia*	1	1.45%
Rotavirus + *G. lamblia*	1	1.45%
Rotavirus + coronavirus	8	11.59%
Coronavirus + *G. lamblia*	5	7.25%
*G. lamblia*	6	8.70%

The prevalence of coinfection among samples was as follows: *C. parvum* and rotavirus in 11.59%; coronavirus and rotavirus in 11.59%; coronavirus and *G. lamblia* in 7.25%; *C. parvum*, coronavirus, and rotavirus in 5.80%; *C. parvum* and coronavirus in 2.90%; rotavirus and *G. lamblia* in 1.45%; and *C. parvum*, coronavirus, rotavirus, and *G. lamblia* in 1.45%.

As seen from the data in Table 2, of the four major pathogens tested, *C. parvum* exhibited a significant preponderance of infection rates relative to the other three and was the predominant pathogen in this infection (*P* < 0.05). There were no significant differences in the proportions among the other three pathogens.

### Detection of Infectious Agent by PCR

#### *C. parvum* 

Subgenotyping data of 69 SSU rRNA sequences was employed to identify *C. parvum*. No novel genotype was obtained by variant BLAST searches. Among 69 samples, 31 (44.93%) were positive for *C. parvum*. Of these samples, 26.09% were infected by *C. parvum* alone, whereas 18.84% were mixed infections. In all mixed infections, *C. parvum* and bovine rotavirus coinfection was predominant (10.14%), followed by coinfection with *C. parvum* and *G. lamblia* (4.35%).

#### Giardia

The primers for β*-giardin* GGL-F/GGL-R (one-step PCR) and the *gdh* gene GDHeF/GDHiRm/GDHiF (two-step nested PCR) failed to amplify all the fecal samples in this study. However, the two sets of β*-giardin* primers G7/G376/G759 (two-step nested PCR) and Gia2029/Gia2150 and RH11/RH4 (two-step nested PCR) successfully amplified five and four samples, respectively. The PCR results showed that all *Giardia* pathogens belong to *G. lamblia*. The percentage of samples positive for *G. lamblia* was 13.04%. The percentage of samples only infected by *G. lamblia* was 5.80%, whereas the percentage of samples coinfected with this species was 7.24%.

#### Bovine Rotavirus

Sequencing identified the bovine rotavirus as bovine rotavirus A. The percentage of samples determined by PCR to be infected with bovine rotavirus was 36.23%. The percentage of samples determined to be coinfected with this virus was 24.64%, whereas the percentage of single-infected samples was 11.59%. Details of mixed infections are shown in Table 4.

**Table 4:** Detection result of infectious agent by PCR.

**Agent**	**Number**	**Percent**
None	16	23.19%
*C. parvum*	18	26.09%
*C. parvum* + bovine rotavirus	7	10.14%
*C. parvum* + bovine coronavirus	1	1.45%
*C. parvum* + *G. lamblia*	3	4.35%
*C. parvum* + bovine rotavirus + bovine coronavirus	2	2.90%
*C. parvum* + bovine rotavirus + bovine coronavirus + *G. lamblia*	1	1.45%
Bovine rotavirus	8	11.59%
Bovine rotavirus + *G. lamblia*	1	1.45%
Bovine rotavirus + bovine coronavirus	6	8.70%
Bovine coronavirus	2	2.90%
*G. lamblia*	4	5.80%

#### Bovine Coronavirus

The overall positive rate of bovine coronavirus by PCR was 17.39% (12/69), and the rate of coinfection was 14.49%. Details of mixed infections are shown in Table 4.

#### *E. coli* 

PCR results for *E. coli* were obtained from 0 (0%) out of 69 fecal samples.

## Discussion

This study investigated and characterized the pathogens causing NCD on cattle farms in Yangxin County, Shandong Province, China. Two rural sample groups were studied, and although a larger sample size would have improved the validity of the results, the sample size for this study was limited by time.

Regarding the characteristics of the farm, the facilities, variety, and number of employees were similar between the two farms. The average size of the two farms was 800 cows, consisting of 80–90% adult cows, making them large-scale dairy farms. Klein-Jobstl believes that the occurrence of diarrhea is significantly associated with herd size (21). These two cattle farms were therefore at greater risk of diarrhea.

The maternity barn is believed to be strongly associated with the health of the calf. Regular cleaning of the calving barns can reduce the occurrence of calf diseases. The time calves spend in the maternity barn with their dams is also a risk factor. In particular, maternity barns have a poor environment with sick animals using maternity areas (22). Most enteric pathogens are transmitted between cows and calves, either by colostrum or by the fecal–oral route of environmental transmission. Even healthy cows carry a large number of pathogens during the perinatal period (23). In addition, adequate intake of colostrum is recognized to improve the immunity of calves and reduce the development of infectious intestinal diseases in calves (24). On the two farms that we investigated, all newborn calves were allowed to suckle colostrum from their dams; therefore, no colostrum-related diarrhea was observed in this investigation.

Barrington recommended housing calves separately, as this approach may result in a lower pathogen exposure (25). In a separate barn, calves are fed on individual basis in line with their specific desires, and it is easier to regulate the animals and identify certain abnormalities. In our survey, calves on both farms were kept in groups indoors with ventilation systems, but environmental hygiene issues were often overlooked, and although relevant disinfection and hygiene protocols were in place on the farms, they were not rigorously followed by the workers.

After calves are weaned from colostrum, their nutrition determines the level of immunity (26). The farms surveyed both used feeding to buy surplus waste milk from elsewhere. For calves, waste milk has a better nutritional composition than concentrated feed and can better enhance the growth of the animal. However, unpasteurized whole milk poses a high risk of intestinal infection if it is not consumed immediately (27). Both farms surveyed established pasteurization units for purchased waste milk to ensure the biosecurity of liquid feed.

A study by Gutzwiller et al. noted that diarrhea was considerably more likely to occur in calves during colder seasons, such as in autumn and winter, than in spring and summer, and that this phenomenon was not related to inadequate levels of immunoglobulin ingested by the calves (24). Our study was conducted in late autumn, and temperatures had started to drop, especially at night. While the change in weather affects calf diarrhea, it also contributes to another important factor—the onset of respiratory disease. There have been many studies demonstrating a close association between respiratory illness and diarrhea (27–29). For herds with >200 cows, the number of routine veterinary visits to the farm and the incidence of respiratory disease in calves accounted for 64.2% of the incidence of calf diarrhea (22). However, due to the short sampling time of the current survey, it was not possible to properly determine the risk of respiratory illness. However, we found that self-grooming of calves can be a risk factor for diarrhea (27). The pathogens become attached to the skin surface of the calf when it makes contact with objects in the barn, such as bars and floors, that may be contaminated with pathogenic bacteria, and then the pathogens from the environment enter the calf's body through licking the skin during self-grooming, leading to diarrhea.

In this investigation, the use of a rapid test kit to detect the four main pathogens of diarrhea demonstrated that *C. parvum* was detected most frequently, which is consistent with the results obtained by Bartels (30). The detection rate of *C. parvum* in the feces of diarrheic calves in our survey was 52.17%. Bartels et al. obtained a detection rate of ~43% for *C. parvum* in 1- to 2-week-old calves with diarrhea and an increase in shedding associated with the use of conventional antibiotics (30). Other studies have reported that over 60% of diarrheic calves are infected with *C. parvum*, either alone or in combination (31, 32). The same study also noted that rotavirus is often mixed with *C. parvum* (33), which is also very similar to the results of the current study (30). Coronaviruses are conditional pathogens that are rarely detected when good management conditions are ensured but can invade the body when environmental conditions become poor. Therefore, coronaviruses generally have a very low detection rate (29, 34). *G. lamblia*, although also a major cause of diarrhea in calves, was detected at a low rate in this survey, as well as in some other surveys (30). In general, *E. coli* is shed in a shorter period, resulting in a lower incidence; therefore, *E. coli* K99 was not detected in this survey (30). Although the use of rapid test kits is convenient and quick, the results obtained with these kits were validated using PCR analyses to verify their accuracy and specificity. The results presented in Table 2 show that there were no significant differences (*P* > 0.05) between the rapid test kit group and the PCR group when they were compared using the chi-square test. This result suggests the feasibility of employing rapid test kit for clinical use in calf diarrhea. Further research to perform pathogenic analysis of diarrheic cattle and ensure the rational use of drugs may be effective in reducing mortality.

## Conclusion

Many factors were determined to be similar between the two farms investigated, including milk management, housing, and feeding procedures. Therefore, no important effects of those factors on the symptom of calves diarrhea were found during this study. In contrast, environmental conditions were shown to be considerably related to calves diarrhea on the farms. Variables that were strongly related to calves diarrhea were farm size, cleanliness of the calving barns during the birth of calves, placement of individual barns, and presence of respiratory disorders. The possible influence of those factors on calves diarrhea on farms should thus be considered. Optimization of these factors may cause a reduction in calves diarrhea, which may thus reduce morbidity and mortality, which would be of economic importance. The use of rapid test kits for the detection of pathogens in diarrheic calves and the administration of appropriate treatment may serve to reduce mortality due to diarrhea on large dairy cattle farms.

## Data Availability Statement

The original contributions presented in the study are included in the article/Supplementary Material, further inquiries can be directed to the corresponding author/s.

## Ethics Statement

The experiment is approved by Research Ethics Committee of Lanzhou Institute of Husbandry and Pharmaceutical Science of CAAS, Lanzhou, PR China. All fecal samples were collected from cattles with diarrhea with the consent of the owners.

## Author Contributions

XW proposed the test scheme. ZD wrote the manuscript and was responsible for revising it. WW and BL performed the laboratory experiments. XZ, FC, and XW collected the samples. JZ provided the funding resource. All authors contributed to the article and approved the submitted version.

## Conflict of Interest

The authors declare that the research was conducted in the absence of any commercial or financial relationships that could be construed as a potential conflict of interest.

## Supplementary Material

Supplementary Figure 1 Rapid kit for positive results.

Supplementary Figure 2 Rapid kit for negative results.
