# Supplementary figures and images for: Detection of Infectious Agents Causing Neonatal Calf Diarrhea on Two Large Dairy Farms in Yangxin County, Shandong Province, China
Source: Front Vet Sci. 2021 Feb 5;7:589126. doi: 10.3389/fvets.2020.589126 (PMC7892430; doi:10.3389/fvets.2020.589126)

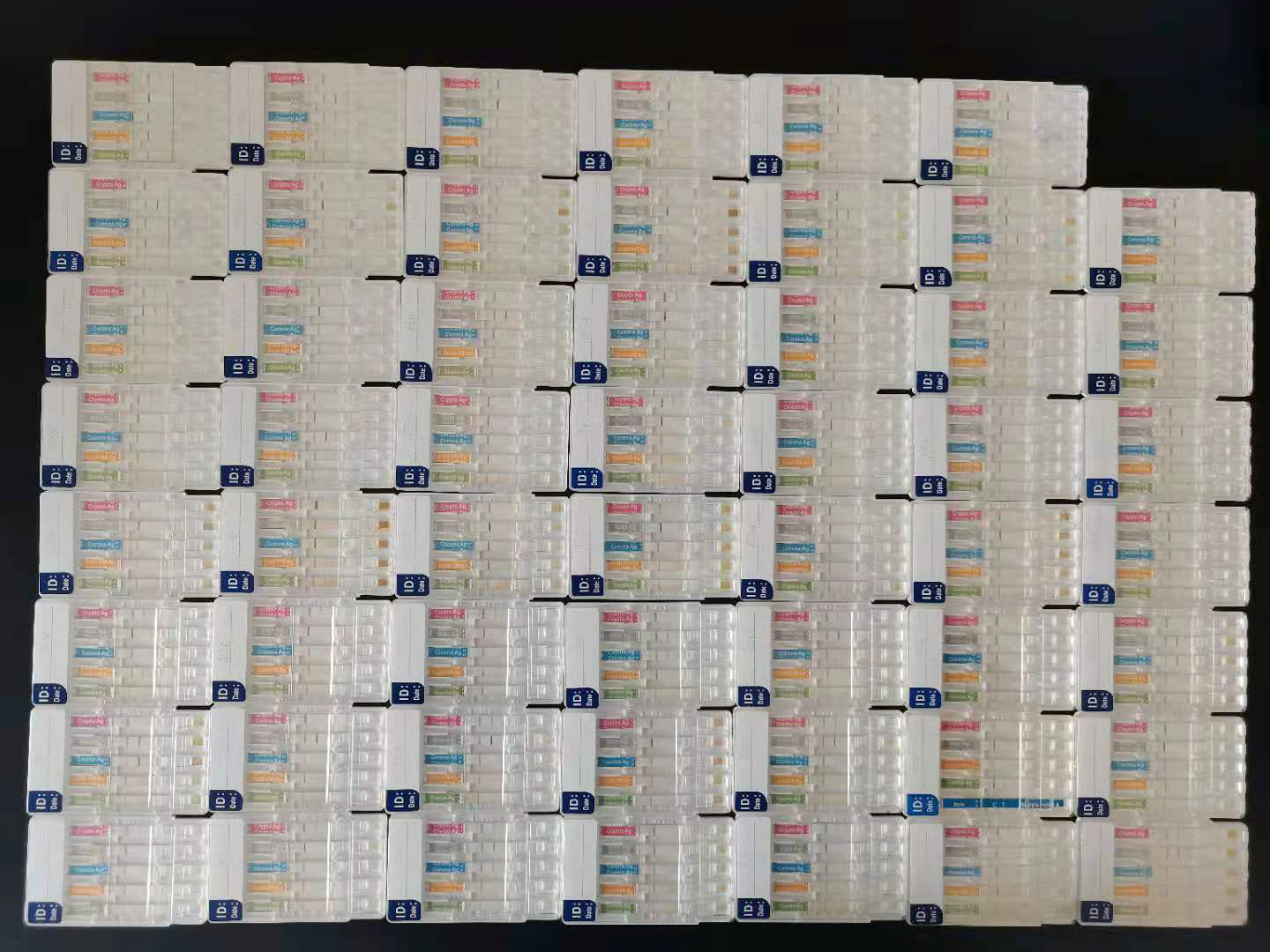

Supplement: Supplementary Figure 1 — Rapid kit for positive results. [file Image_1.TIF]

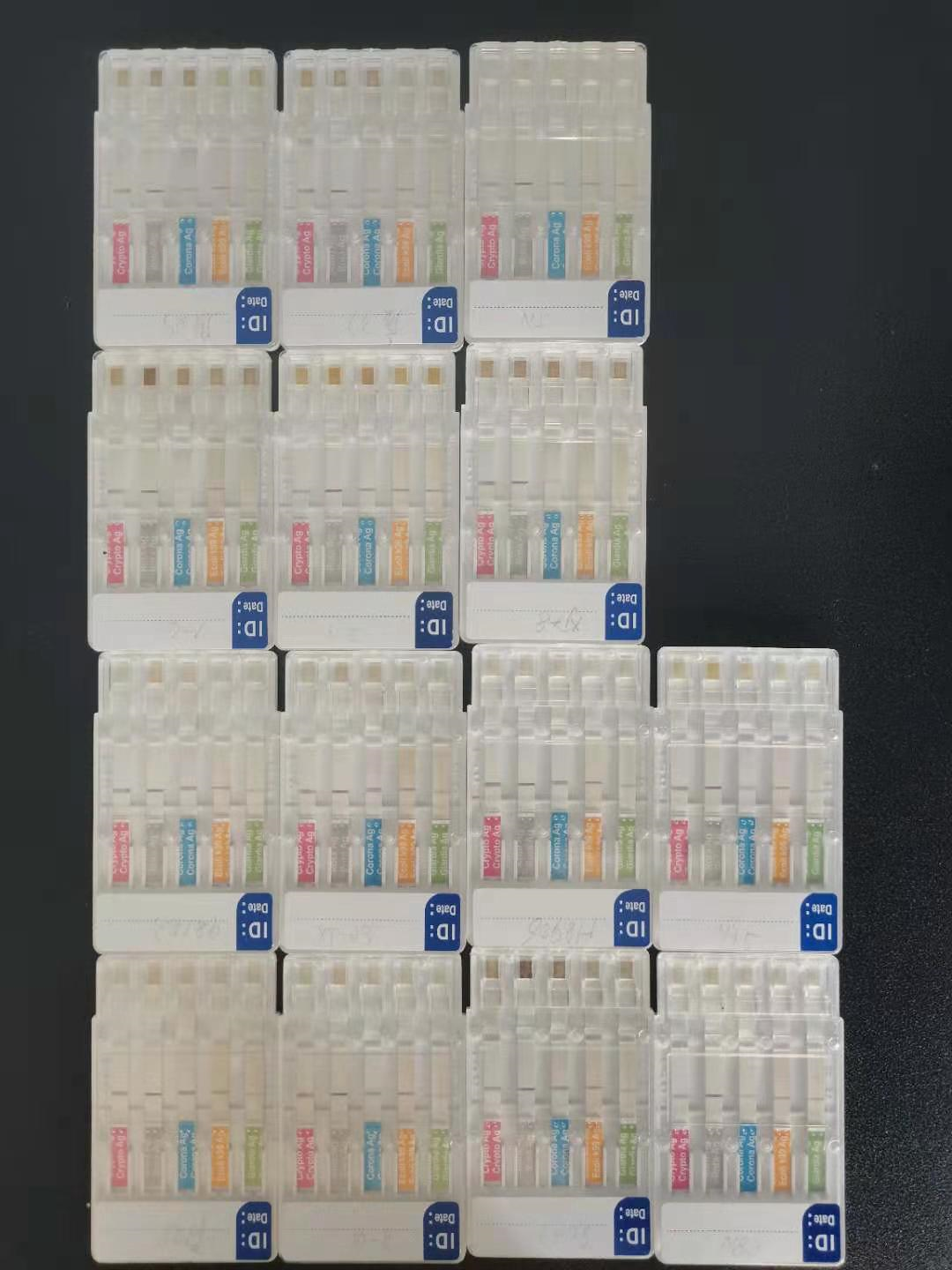

Supplement: Supplementary Figure 2 — Rapid kit for negative results. [file Image_2.TIF]
